# Impact of Dose Titration Using the Seizure Quality Rating (SQR) Scale on Electroconvulsive Therapy (ECT) Outcomes: A Quality Improvement Project

**DOI:** 10.1192/bjo.2025.10409

**Published:** 2025-06-20

**Authors:** Mohsin Nazeer Muhammed, Raja Badrakalimuthu

**Affiliations:** Surrey and Borders Partnership NHS Foundation Trust, Surrey, United Kingdom

## Abstract

**Aims:** This Quality Improvement Project (QIP) aimed to assess the impact of incorporating the Seizure Quality Rating (SQR) scale into ECT dose titration practices. The objective was to optimize treatment by improving seizure quality assessment and reducing cognitive side effects associated with higher electric doses, ultimately enhancing patient outcomes.

**Methods:** The QIP was conducted at the ECT clinic of Surrey & Borders Partnership NHS Foundation Trust. The SQR scale evaluated seizure quality using parameters such as EEG and visual seizure duration, mid-ictal amplitude, interhemispheric coherence, postictal suppression, and peak heart rate. Patients were categorized into two groups:

Pre-SQR (Standard Group): Treated between May 1, 2023, and October 30, 2023, with dose titration based on seizure duration.

SQR (Post-Intervention Group): Treated between December 1, 2023, and May 31, 2024, with dose titration guided by the SQR scale.

Inclusion criteria were depressive disorders (e.g. recurrent depression, bipolar depression), excluding primary psychotic disorders. Outcomes measured included the number and dose of treatments, Clinician’s Global Improvement (CGI) scores, and subjective memory reports (patient-rated).

**Results:** The SQR group (n=7) received lower mean electric doses (622 mC) compared with the pre-SQR group (n=11; 705 mC) while maintaining comparable therapeutic outcomes. Both groups required a similar number of treatments (mean: 12 sessions). CGI improvement scores (CGI-I) and subjective memory ratings post-ECT showed no significant difference between the groups. These results suggest that the SQR scale may support safer dosing without compromising clinical efficacy.

**Conclusion:** The integration of the SQR scale into ECT practice demonstrated a promising trend toward optimizing treatment by reducing electric doses while maintaining clinical effectiveness. This structured approach offers the potential to minimize cognitive side effects, particularly in vulnerable populations such as the elderly. These findings underline the broader implications of incorporating data-driven tools like the SQR scale into routine ECT protocols across various trusts to enhance precision, safety, and patient outcomes on a wider scale. Future recommendations include gathering patient feedback to assess the cognitive and mood benefits of lower doses, conducting further research with larger cohorts to validate the findings, and embedding the SQR scale into standard ECT guidelines to promote consistency and improve treatment outcomes.

